# Thrombin Generation in the Glasgow Myocardial Infarction Study

**DOI:** 10.1371/journal.pone.0066977

**Published:** 2013-06-24

**Authors:** Machiel Smid, Arne W. J. H. Dielis, Henri M. H. Spronk, Ann Rumley, Rene van Oerle, Mark Woodward, Hugo ten Cate, Gordon Lowe

**Affiliations:** 1 Laboratory of Clinical Thrombosis and Haemostasis, Department of Internal Medicine, Cardiovascular Research Institute Maastricht, Maastricht University, Maastricht, The Netherlands; 2 Institute of Cardiovascular and Medical Studies, University of Glasgow, Glasgow, United Kingdom; 3 George Institute, University of Sydney, Sydney, Australia; Innsbruck Medical University, Austria

## Abstract

**Background:**

Thrombin is a key protease in coagulation also implicated in complex pathology including atherosclerosis. To address the role of thrombin in relation to myocardial infarction (MI) we explored thrombin generation analysis in plasma from patients and controls that had participated in the Glasgow MI Study (GLAMIS).

**Methods:**

Thrombin generation at 1 and 2 pM TF and with and without thrombomodulin (TM) was performed on plasmas from 356 subjects (171 cases, 185 age and sex matched controls) from GLAMIS collected between 3 and 9 months after the MI event.

**Results:**

Although thrombin generation was slightly delayed in cases (lag time increased from 3.3 to 3.6 min) at the highest trigger, the overall potential to generate thrombin was increased by 7% for the ETP and by 15% for the peak height (both at the 1 pM TF trigger) in cases. Addition of TM did not reveal differences. Furthermore, an increased thrombin generation was associated with MI [normalized ETP: adjusted OR for the highest percentile = 2.4 (95% CI 1.3–4.5) and normalized peak height: adjusted OR = 2.6 (1.3–5.0)] at the lowest trigger; normalized ETP and peak height being 2.1 (1.1–3.8) and 2.0 (1.0–4.1) at the higher 2 pM trigger.

**Conclusion:**

In GLAMIS, patients with a previous MI had an increased thrombin generation compared to controls. The absence of a clear difference in TM reduction suggests an unaltered anticoagulant activity in these patients. Further research is needed in order to unravel the underlying mechanisms of enhanced thrombin generation after MI.

## Introduction

In the pathogenesis of acute myocardial infarction (MI) common risk factors for atherosclerosis as well as hemostatic factors are important determinants. Thrombin is a central enzyme in the blood coagulation cascade, regulating platelet activity and fibrin clot formation. In addition, thrombin appears to modulate atherosclerosis formation and plaque phenotype, at least in experimental studies [1]. Clinically, markers of thrombin generation have been linked to risk of recurrent arterial thrombosis, but the evidence is still weak [2]. We recently showed that the level of thrombin generation in plasma was elevated during and following a first MI [3]. Contra-intuitively, endogenous thrombin potential (ETP), one of the main parameters derived from thrombin generation analysis, tended to be lower in those who had a subsequent recurrent arterial vascular event, which in conjunction with an elevated level of D-dimer yielded a significant risk for recurrence of 5.8 [3]. These analyses were however confined to plasma samples collected during the acute phase of AMI. Since *in vitro* thrombin generation analysis is typically aimed at establishing the *potential* to generate thrombin in a given plasma aliquot, rather than the actual amount of thrombin formed *in vivo* in the acute phase, we set out to compare thrombin generation in plasma samples collected in individuals with previous myocardial infarction and matched controls, collected in the Glasgow Myocardial Infarction Study (GLAMIS). Since, in patients with stable coronary artery disease we expected only modest differences in clotting potential we chose to stimulate plasma with low levels of tissue factor, ie the commonly used 1 pM as well as a slightly higher 2 pM tissue factor concentration. We calculated odds ratios for myocardial infarction based on *in vitro* thrombin generation data, in order to assess the importance of thrombin formation in plasma, in the absence of platelets, as a risk factor for coronary artery disease.

## Methods

As described in previous papers [4], [5] GLAMIS was established to investigate associations of plasma haemostatic and inflammatory variables with previous myocardial infarction (MI) and conventional risk factors in a case-control study. The aim was to recruit all men and women with MI in the North Glasgow MONICA study [6] diagnosed by W.H.O. - MONICA criteria from July 1994, between 3 and 9 months after the event, minimizing acute phase influences. Cases were patients with MI in this population survey who were still alive and willing to give consent (75% response rate). Controls were selected from a random sample of the same north Glasgow population, obtained from general practice registers, and frequency matched for sex and age (within 1 year), who had no history or electrocardiogram evidence of MI. Written informed consent was obtained from all participants, and the study was approved by the local Research Ethics Committee: Greater Glasgow Health Board Research Ethics Committee. Participants completed a general health questionnaire and blood pressure was recorded. A forearm venous sample was taken after a full overnight fast and care was taken to have properly collected blood samples in all individuals. Lipid assays were measured as previously described [4]. Venous blood was anticoagulated with trisodium citrate (3.2%(w/v)) and centrifuged at 2000×g for 10 min at room temperature within 2 h of sampling, and aliquots were stored at −70°C until assay. For these analyses only previously unthawed plasma samples were used.

### Thrombin Generation Assay

One of the methods developed for studying *in vitro* thrombin generation in plasma is the Calibrated Automated Thrombogram (CAT: Thrombinoscope BV, Maastricht, The Netherlands). This assay is based on the principle that rather than assessing the coagulation status at a single timepoint, the potential to clotting could reveal the actual risk of clotting under pathologic conditions, eg plaque rupture. To this end plasma is usually triggered with small amounts of tissue factor (TF) and the thrombin generation curve is measured in time. By adding thrombomodulin also the influence of the natural anticoagulant protein C system can be addressed. Briefly, *in vitro* thrombin generation in platelet-poor plasma was measured by means of the CAT method (Thrombinoscope BV, Maastricht, the Netherlands), which employs a low affinity fluorogenic substrate for thrombin to continuously monitor thrombin activity in plasma. Measurements were conducted in 80 µL platelet-poor plasma in a total volume of 120 µL (20 µL fluorogenic substrate, calcium and 20 µL solution of TF and phospholipids). Final concentrations of TF were 1 (PPP Reagent Low) and 2 pM TF (kind gift of Thrombinoscope BV) in the presence of 4 µM phospholipids (phosphatidylserine/phosphatidylcholine/phosphatidylethanolamine vesicles in HEPES-buffered saline). Since we anticipated that differences in the thrombin generating potential would be modest between cases and controls under non-acute conditions, we chose two low stimuli of plasma: 1 and 2 pM of TF [3]. Although this would theoretically pose a risk of being susceptible to contact activation, based on our previous data we did not expect much contact activity influence at TF concentrations >1 pM [7]. In order to have a trigger that would be a little more robust we also tested a 2 pM TF concentration.

To assess the contribution of the protein C pathway on *in *vitro thrombin generation, all measurements were conducted in the absence and presence of thrombomodulin (TM), titrated to obtain 50% reduction in ETP of normal pooled platelet poor plasma [8]. In order to correct for inner-filter effects and substrate consumption, each thrombin generation measurement was calibrated against the fluorescence curve obtained in the same plasma to which a fixed amount of thrombin-α2-macroglobulin complex was added (Thrombin Calibrator, Thrombinoscope BV). Fluorescence was read in a Fluoroskan Ascent reader (Thermo Labsystems OY, Helsinki, Finland) equipped with a 390/460 nm filter set and thrombin generation curves were calculated with Thrombinoscope software (Thrombinoscope BV). Three parameters were derived from the thrombin generation curves: lag time (initiation phase of coagulation), endogenous thrombin potential (ETP; area under the thrombin generation curve), and peak height. Within and between run coefficients of variations for *in vitro* thrombin generation were below 10% for all parameters derives, as described previously [9].

### Statistical Analysis

Thrombin generation data for lag time, normalized ETP (nETP), normalized peak height (nPeak Height) and reduced ETP were not normally distributed and are thus presented as median [95% CI]. Differences between cases and controls were calculated using the Mann-Whitney U test.

Thrombin generation parameters were categorized based on the cut-off values of the highest 10% in the control group. Logistic regression was used to obtain odds ratios (ORs) and corresponding 95% confidence intervals (95% CI) as a measure of relative risk with the lowest category as a reference category. Analyses were adjusted for the stratification factors: hypertension (defined as diastolic blood pressure >90 mmHg and/or systolic blood pressure >140 mmHg), BMI, hyperlipidemia (defined as total cholesterol >6 mmol/L), diabetes mellitus, (history of) smoking, and gender. All statistical analyses were performed using SPSS version 19. P<0.05 was considered statistically significant.

## Results

The present analysis includes 171 cases and 185 controls. Baseline characteristics are indicated in Table 1, showing significantly more unfavorable risk factors and biochemical parameters in the cases compared to the controls, except for both systolic and diastolic blood pressure, which were higher in the control group. With regard to medication, we excluded any users of oral anticoagulants, because of its known inhibitory effect on thrombin generation. Aspirin and statins were used in 95% and 18% of cases and in 12 and 2% of controls, respectively.

**Table 1:** Baseline characteristics.

		Cases		Controls		p
		(n = 171)		(n = 185)		
**Age**	**(years)**	54.4	(7.7)	54.2	(7.7)	0.86
**Gender**	**(male, n(%))**	126	(73.7)	137	(74.1)	0.94
***Risk factors***						
**Smoking**	**(n, (%))**					
** Ex and current**		153	(89.5)	136	(73.5)	
** Never**		16	(9.4)	49	(26.5)	**<0.01**
**Diabetes**	**(n, (%))**	18	(10.5)	3	(1.6)	**<0.01**
**Blood pressure**						
** Systolic**	**(mmHg)**	124.6	(22.0)	129.6	(21.0)	**0.06**
** Diastolic**	**(mmHg)**	79.4	(13.8)	81.9	(11.3)	**0.03**
**BMI (kg/m^2^)**	**(kg/m^2^)**	28.0	(5.0)	26.6	(4.3)	**<0.01**
**Lipid profile**						
** Total Cholesterol**	**(mmol/L)**	6.0	(1.2)	5.8	(1.0)	**0.03**
** HDL**	**(mmol/L)**	1.1	(0.3)	1.4	(0.4)	**<0.01**
** Triglycerides**	**(mmol/L)**	2.2	(1.5)	1.8	(1.5)	**<0.01**

Data are presented as mean (standard deviation).

### Thrombin Generation

**Figure 1:**
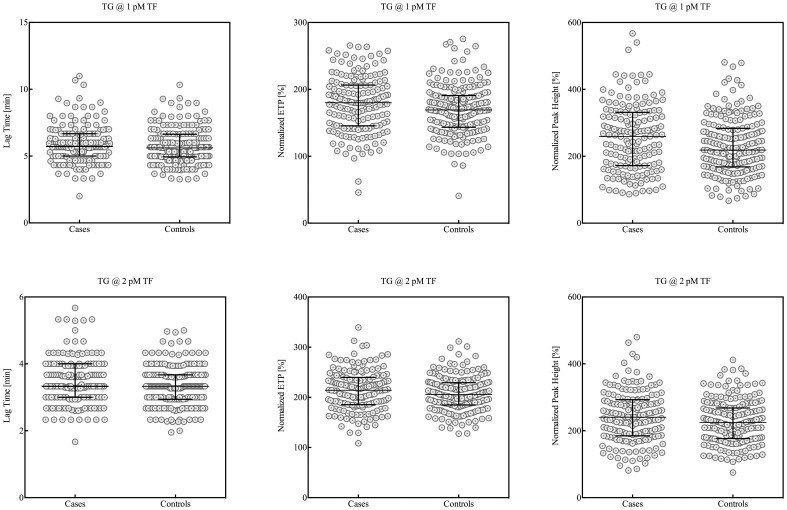
Thrombin generation triggered with either 1 or 2 pM tissue factor (TF) in cases and controls.

Table 2 and Figure 1 show thrombin generation parameters for cases and controls. The ETP and peak height were normalized against pooled plasma from healthy donors in order to correct for between run variance, as reported previously [9]. Although not significant, thrombin generation triggered with 1 pM TF was delayed in cases as indicated by a prolongation of the lag time from 5.6 min for controls to almost 6.0 min (p>0.05) for cases, whereas the overall potential to generate thrombin was significantly increased in cases. Both the normalized ETP (180% [145–206]) and peak height (245% [170–324]) were enhanced in cases compared to controls (ETP: 168% [143–189], peak height: 213% [166–279], p<0.05). Prolongation of the lag time (3.61 min [3.00–4.00] vs. 3.33 min [2.94–3.67], p<0.05) for cases was confirmed by thrombin generation triggered with 2 pM TF. At this higher TF trigger, the normalized ETP and peak height were both higher in cases than in controls but the difference was not statistically significant. At both TF triggers, the addition of thrombomodulin did not reveal any differences in reduction of the ETP between cases and controls (Table 2).

**Table 2:** Thrombin generation.

		*Cases*		*Controls*		*p*
@ *1 pM TF*		*(n = 171)*		*(n = 185)*		
** Lag Time**	(min)	5.94	(5.00–6.67)	5.61	(4.67–6.67)	n.s.
** nETP**	(%)	180	(145–206)	168	(143–189)	**0.023**
** nPeak Height**	(%)	245	(170–324)	213	(166–279)	**0.017**
** reduced ETP**	(%)	−33	(−20– −43)	−36	(−22– −46)	n.s.
@ ***2 pM TF***						
** Lag Time**	(min)	3.61	(3.00–4.00)	3.33	(2.94–3.67)	**0.025**
** nETP**	(%)	213	(184–239)	204	(184–227)	n.s.
** nPeak Height**	(%)	236	(183–290)	221	(177–267)	n.s.
** reduced ETP**	(%)	−41	(−27– −53)	−43	(−32– −56)	n.s.

Data are presented as median [IQR] for lag time, normalized ETP (nETP), normalized peak height (nPeak Height) and the reduction on ETP upon addition of thrombomodulin (reduced ETP). Thrombin generation was assessed at a trigger of 1 or 2 pM tissue factor (TF) in the presence of 4 µM phospholipids, by means of the Calibrated Automated Thrombogram.

Thrombin generation parameters showed statistically significant correlations with some of the classical risk factors for myocardial infarction, including BMI, cholesterol and triglycerides (Table 3). However, almost all correlations were weak (Rs<0.35) and not always consistent between the 1 and 2 pM TF trigger (HDL). All risk factors (age, BMI, hypercholesterolemia, hypertension, diabetes and gender) were included in the multivariate analysis.

**Table 3:** Correlation coefficients (Rs) between common risk factors for myocardial infarction and thrombin generation parameters.

	Lag Time		nETP		nPeak Height		reduced ETP	
	r	p	r	p	r	p	r	p
@ **1 pM TF**								
**Age**	−0.053	(0.318)	0.043	(0.419)	0.033	(0.537)	**0.139**	(0.009)
**Blood pressure**								
** Systolic**	0.012	(0.818)	0.096	(0.070)	0.055	(0.303)	0.014	(0.791)
** Diastolic**	0.082	(0.123)	0.093	(0.080)	0.040	(0.455)	−0.072	(0.178)
**BMI**	**0.140**	(0.009)	**0.265**	(0.000)	**0.216**	(0.000)	0.083	(0.120)
**Lipid profile**								
** Total Cholesterol**	**0.227**	(0.000)	**0.134**	(0.011)	0.056	(0.298)	−0.083	(0.119)
** HDL**	**−0.240**	(0.000)	**−0.114**	(0.032)	−0.071	(0.186)	−0.038	(0.477)
** Triglycerides**	**0.273**	(0.000)	**0.210**	(0.000)	**0.211**	(0.000)	0.084	(0.116)
@ **2 pM TF**								
**Age**	−0.010	(0.848)	0.037	(0.486)	0.062	(0.240)	**0.170**	(0.002)
**Blood pressure**								
** Systolic**	0.059	(0.267)	0.044	(0.405)	−0.004	(0.945)	0.004	(0.941)
** Diastolic**	0.064	(0.227)	0.062	(0.243)	−0.033	(0.532)	−0.083	(0.127)
**BMI**	**0.181**	(0.001)	**0.283**	(0.000)	**0.185**	(0.000)	0.027	(0.614)
**Lipid profile**								
** Total Cholesterol**	**−0.248**	(0.000)	**0.110**	(0.032)	−0.016	(0.436)	**−0.200**	(0.000)
** HDL**	**0.248**	(0.000)	**0.114**	(0.039)	−0.042	(0.758)	0.020	(0.710)
** Triglycerides**	**0.323**	(0.000)	**0.117**	(0.001)	0.084	(0.114)	−0.086	(0.113)

nETP: normalized endogenous thrombin generation, nPeak Height: normalized peak height. Data are presented as R (p-value).

Table 4 shows the odds ratios (ORs) for myocardial infarction for the different thrombin generation parameters, triggered with both 1 and 2 pM tissue factor. Unadjusted ORs for myocardial infarction with increasing thrombin generation parameters at 1 pM of TF trigger were 2.7 (95% CI 1.4–4.9) for the normalized ETP and 2.5 (95% CI 1.3–4.6) for the normalized peak height for cases in the highest 10^th^ percentile. ORs for the lag time and ETP reduction in the presence of TM were not statistically significant (Table 4). Analysis of thrombin generation parameters obtained with the 2 pM tissue factor trigger revealed essentially similar ORs as those for 1 pM TF (Table 4).

**Table 4:** Distribution of thrombin generation parameters and odds ratios for myocardial infarction.

							Adjusted	
	Patients		Controls		OR	(95% CI)	OR*	(95% CI)
	(n = 171)		(n = 185)					
@ **1 pM TF**								
**Lag Time**								
** <7.66**	146	(85)	167	(90)	1		1	
** >7.66**	25	(15)	18	(10)	1.6	(0.8–3.0)	1.225	(0.6–2.4)
**nETP**								
** <211**	133	(78)	167	(90)	1		1	
** >211**	38	(22)	18	(10)	2.7	(1.4–4.9	2.4	(1.3–4.5)
**nPeak Height**								
** <336**	135	(79)	167	(90)	1		1	
** >336**	36	(21)	18	(10)	2.5	(1.3–4.6)	2.6	(1.3–5.0)
**Reduced ETP**								
** < −14**	145	(85)	167	(90)	1		1	
** > −14**	26	(15)	18	(10)	1.7	(0.9–3.2)	1.7	(0.8–3.3)
@ **2 pM TF**								
**Lag Time**								
** <4.33**	153	(89)	167	(90)	1		1	
** >4.33**	18	(11)	18	(10)	2.1	(0.9–4.6)	2.0	(0.9–4.7)
**nETP**								
** <248**	140	(82)	167	(90)	1			
** >248**	31	(18)	18	(10)	2.1	(1.1–3.8)	2.1	(1.1–4.0)
**nPeak Height**								
** <311**	141	(82)	167	(90)	1		1	
** >311**	30	(18)	18	(10)	2.0	(1.1–3.7)	2.0	(1.0–4.1)
**Reduced ETP**								
** < −20**	136	(80)	162	(88)	1		1	
** > −20**	27	(16)	17	(9)	1.9	(1.0–3.6)	1.8	(0.8–3.7)

Ref, reference category; 95% CI, 95% confidence interval. OR are relative to the reference category. OR* adjusted for stratifying factors (hypertension, BMI, hyperlipidemia, diabetes mellitus, history of smoking and gender) at the time of blood sampling. Thrombin generation was assessed at a trigger of 1 or 2 pM TF in the presence of 4 µM phospholipids. Thrombin generation parameters included lag time, normalized ETP (nETP), normalized peak height (nPeak Height) and the reduction on ETP upon addition of thrombomodulin (reduced ETP).

Adjustment for putative confounders (age, gender, BMI, hypertension, smoking, diabetes, hypercholesterolemia) attenuated the OR for the normalized ETP to 2.4 (95% CI 1.3–4.5) and enhanced the OR for the normalized peak height to 2.6 (95% CI 1.3–5.0), both assessed with the 1 pM TF trigger. At 2 pM TF, adjusted ORs for myocardial infarction for the normalized ETP and peak height were comparable to the 1 pM condition showing increased OR’s for normalized peak and ETP in cases versus controls (Table 4). The use of aspirin or statins did not significantly affect any of the thrombin generation variables (data not shown).

## Discussion

The present data show that in patients with previous myocardial infarction *in vitro* thrombin generation in plasma is altered as compared to control individuals, showing a prolonged lag time (at the 2 pM tissue factor trigger only) and an increased normalized ETP and peak height (albeit at the 1 pM TF concentration only). Moreover, those cases with the highest levels of thrombin generation (normalized ETP and peak height values) also have an increased risk of MI, with adjusted OR’s of between 2 and 2.6, for the different test conditions. Taken together, these data suggest that the sustained increased thrombin generation potential is a pathophysiologically relevant characteristic and indicator of a hypercoagulable state in plasma from patients with coronary disease. The prolonged lag time in cases of the present study is in accordance with the observation in the MARK study, where lagtime was prolonged in the acute phase but also at later timepoints [3]. This prolongation may be due to release of TFPI from perturbed endothelium such as demonstrated in a recent case control study in young women with manifestations of arterial thrombosis (AMI or stroke) [10]. However, also other changes in the balance between pro- and anticoagulant proteins may be involved in the alterations in thrombin generation profile.

Although there appears to be an effect of common cardiovascular risk factors including BMI, cholesterol and triglycerides on the plasma phenotype, such effects were modest at best in the present analysis. The effect of BMI was recently found to be due to total body fat in an analysis of the Hoorn study [11] and probably mediated by inflammation. The effect of cholesterol and triglyceride has also been postulated to influence coagulation in earlier studies [12]. Obviously, there is also the possibility of unrecognized confounding.

The use of medication that might affect thrombin generation, including aspirin and statins did not affect any of the thrombin generation variables. For aspirin an effect in platelet poor plasma is not to be expected, although one recent report indicates a trend for accelerated thrombin formation in long-term aspirin users [13]. The use of statins might have attenuated thrombin generation in platelet poor plasma, as reported in several studies [14]–[16]. The fact that we did not observe any effects may have been due to the relatively low percentage of statin users in the present study.

The present data provide circumstantial support for a causal association between hypercoagulability and arterial vascular disease, as can be postulated on the basis of extensive experimental data [1]. Increased thrombin production may add to the risk of thrombosis in conditions, such as atherosclerosis, when the anticoagulant reserve may be limited and erosion or rupture of the atherosclerotic plaque cannot be properly compensated, resulting in atherothrombosis. It is uncertain which mechanisms link cardiovascular risk factors to thrombin generation, but inflammation driven tissue factor production within the vasculature may be a common mechanism. Inflammatory activity can trigger hypercoagulability in various ways, involving cellular and plasma compartments. In our analysis we only observe effects in platelet poor plasma, which however is the product of interactions with vessel wall and other cellular mediated reactions. It is attractive to consider the plasma coagulation proteome, studied in the *in vitro* thrombin generation assay, as a product of the bidirectional interplay between clotting, vessel wall and inflammatory systems. This may explain occasional “paradoxical” outcomes as prolonged lag time or reduced ETP and peak height [3] in patients in the *acute* phase of coronary artery disease, where counteracting forces (including TFPI) may delay but not impair thrombin generation in plasma.

Several comments regarding the study design and interpretation of outcomes should be made. First, MI was defined according to the WHO-MONICA criteria from 1994, which may have resulted to greater heterogeneity in the case selection than would have occurred according to current criteria. Second, inherent to the study design, where blood sampling in the cases took place three to nine months after the MI no firm conclusions can be drawn on causality. The possibility of reverse causation should indeed be considered. Besides, the diagnostic value of *in vitro* thrombin generation analysis remains to be established. Given the substantial overlap in thrombin generation values between cases and controls, it is unlikely that thrombin generation analysis will provide a diagnostic or prognostic tool for coronary disease. The main advantage of this and similar studies is to obtain better insight in the contribution of plasmatic hypercoagulability to cardiovascular disease, which may ultimately help to identify patients at high risk of fibrin clot formation.

As expected in this case-control design, the case group shows more unfavorable risk factors for cardiovascular disease than the control group, although blood pressure was lower in the cases. This probably reflects blood pressure lowering therapy as secondary prevention after the MI.

A limitation of the study is the significant time of storage of plasma samples until assay, a potential problem in all studies relying on stored plasma material. However, any measurement error incurred by long-term storage would lead to underestimation of the case-control differences. While limited literature [17], [18] suggests there may be some loss in factor VII and factor VIII activities in stored samples over time there is no good reason to expect that, other than a systemic effect on all plasma determinations, this would alter the differences between cases and controls. Nevertheless, the potential of storage associated effects on the laboratory data cannot be excluded. A confounding effect of contact activation at these rather low TF triggers cannot be fully excluded, however, one would have expected a shortening of lag time in case of a significant contribution of contact driven thrombin generation. These test conditions were chosen in order to make the test sufficiently sensitive to the expected, modest differences in thrombin generation that might have escaped detection at the most common 5 pM TF concentration. The downside may be that the TG analysis becomes more sensitive to artificial contact activation [7], [19], [20]. In spite of a certain uncertainty about contact activation interference, there is no good reason to suspect that a random effect of contact activation would contribute to a systematic difference between values in cases and controls. Therefore, we conclude that the observed differences are patient related in cause and not due to pre-analytical artifacts.

In conclusion, we report sustained increases in thrombin generation three to nine months after a MI. Whether this hypercoagulable state indicates specific patients at risk of arterial thrombosis, due to increased plasma based clotting activity, posing a risk factor for recurrent cardiovascular disease, should be the topic of further research.
